# Prevalence of gastrointestinal nematode parasites of dogs and associated risk factors in Gondar town, Northwest Ethiopia

**DOI:** 10.1016/j.heliyon.2024.e41174

**Published:** 2024-12-14

**Authors:** Tsedalu Yirsa, Amare Bihone, Getenew Teshager, Yimer Muktar, Abebe Berihun

**Affiliations:** Department of Veterinary Medicine, College of Agriculture, Woldia University, Ethiopia

**Keywords:** Dogs, Gondar, Nematode parasites, Prevalence, Risk factors

## Abstract

Intestinal parasites commonly infect dogs and can potentially spread to humans globally. Regrettably, numerous dog owners do not give priority to managing their pets' health, often ignoring treatment unless the animal is already ill. Thus, a cross-sectional study was conducted from December 2022 to April 2023 in Gondar town to determine the prevalence of gastrointestinal nematode parasites in dogs and their associated risk factors. A total of 204 dogs stool samples were collected using purposive sampling techniques. Direct fecal smear and flotation techniques were also used to identify the parasite ova. Chi-square and logistic regression were used to analyze the occurrence of the parasite within the risk factors. The overall prevalence of gastrointestinal nematodes in dogs was 47 (23.03 %) from the total 204 dog stool samples. Among these, *Ancylostoma* (9.31 %) was the most gastrointestinal parasite observed followed by *Toxocara*, *Strongyloidea,* and *Trichurius*. The dog positivity in gastrointestinal nematode occurrence had a statically significant association between age, sex, body condition, and housing management (*P-value* ≤ 0.05). Female dogs were 4.5 times (COR: 4.55) and young dogs were 3.5 times (COR: 3.5) more likely exposed to these parasites than their respective male and adult dogs. Regarding body condition scores, poor were 6.5 times (COR: 6.55; 95 % CI: 0.06–0.56) and medium was 0.5 times (COR: 1.5; 95 % CI: 0.26–0.9 9) more exposed than their comparable good body conditions. Generally, this finding suggests potential public health hazards associated with low levels of nematode infections in dogs.

## Highlights

• Direct fecal smear and flotation techniques were also used to identify the parasite ova.
• The overall prevalence of gastrointestinal nematodes in dogs was 47 (23.03 %) from the total examined 204 dog stool samples.
• *Ancylostoma* (9.31 %) was the most gastrointestinal parasite observed followed by *Toxocara*, *Strongyloidea,* and *Trichurius.*
• Female and young age dogs were significantly exposed to these gastrointestinal nematodes.
• Generally, it is crucial to raise awareness about preventive measures and management tactics for these parasites.

## 1 Introduction

Dogs represent the most well-adapted canid species to coexist with humans globally [[1], [2], [3]]. They are closely associated with humans providing companionship, security and protein [4,5]. It is also kept as a pet and companion and serves a range of cultural, social and economic functions in the society [6,7]. However, dogs also carry the most common zoonotic nematode pathogens such as *Toxocara canis* (*T. cani*) and *Ancylostoma caninum* (*A. caninum*) [8]. *Toxocara canis* transmission also occurs both transplacentally and transmammary, primarily detected in puppies. Puppies typically have an ascarid infection from birth or at a young age [6,9]. Dogs may display different clinical symptoms of parasitic infestation, and sometimes an infected dog might not exhibit any signs. Inadequate hygiene practices also heighten the risk of these animals acquiring zoonotic diseases [7].

*Toxocara canis* can cause diarrhoea, poor growth, and death if present in large numbers in puppies [9]. *Ancylostoma caninum* is also one of the most pathogenic nematode parasites in dogs, especially puppies. These nematodes are hematophagous and can cause anaemia and death if present in large numbers [10]. Canine and human infection with zoonotic canine *Anchylosoma* and *Toxocara* can occur through ingestion of the infective eggs and ingestion or skin penetration of the infective larvae [9]. It is also transmitted via food, water, and soils contaminated with dog excreta or secretions, and/or consumption of dog meat [4].

Human infection with *T. cani* is typically asymptomatic; however, some individuals develop visceral larva migrans and ocular *Toxocarias*is [11]. *Ancylostoma* species are the etiological agents of cutaneous larva migrans [6] and *A. caninum* has also been associated with eosinophilic enteritis in humans [12,13]. Therefore, it is important to understand the epidemiology of the nematode parasites of dogs to improve animal health and prevent zoonotic transmissions that cause human sickness and death [14]. Environmental contamination with dog feces harbouring various infective stages of parasites such as eggs, larvae, or assets acts as a leading source of infection of livestock and humans [[15], [16], [17]]. These diseases cause direct and indirect losses to the health of humans and their animals. The prevalence of nematode parasites has been shown to vary considerably from one geographic region to another depending on the GIT nematode involved, the animal species, and local environmental conditions such as humidity, temperature, rainfall, vegetation, and management practices [4,18,19]. The chronic prevalence and spread of these illnesses in less developed nations have been linked to inadequate hygienic practices, insufficient veterinary care for parasitic zoonotic diseases in dogs, and low public awareness of the illnesses' existence and spread [3]. Many stray dog's dog-managed schemes harbour various diseases in different parts of Ethiopia [14].

Gastrointestinal nematode parasites in livestock have been extensively studied in various parts of Ethiopia [[20], [21], [22]]. Nevertheless, there have been limited previous studies on gastrointestinal nematode worms in dogs across different regions of Ethiopia [4,5,14,[23], [24], [25]]. The investigation into the presence of numerous gastrointestinal nematode parasites in dogs has been minimal and has received little attention in Ethiopia [23]. Furthermore, there are limited recent studies on the prevalence of nematode infection in dogs in Gondar [2]. The associated risk factors that predispose dogs to these nematode worms in the study area have received extremely little attention. The different agro climates and diverse cultural practices of the people may influence the coexistence with dogs and the limited use of dog-deworming practices [2,3,26]. Regular monitoring of the prevalence of these parasites in a specific area is crucial for the successful development and execution of an effective worm control strategy [27]. Therefore, this study aimed to establish the prevalence of gastrointestinal nematode parasites in dogs and identify their associated risk factors in Gondar town, Northwest Ethiopia. This information was crucial for implementing appropriate nematode worm control and prevention measures in areas where dogs are raised.

## 2 Materials and methods

### 2.1 Description of the study area

**Fig. 1:**
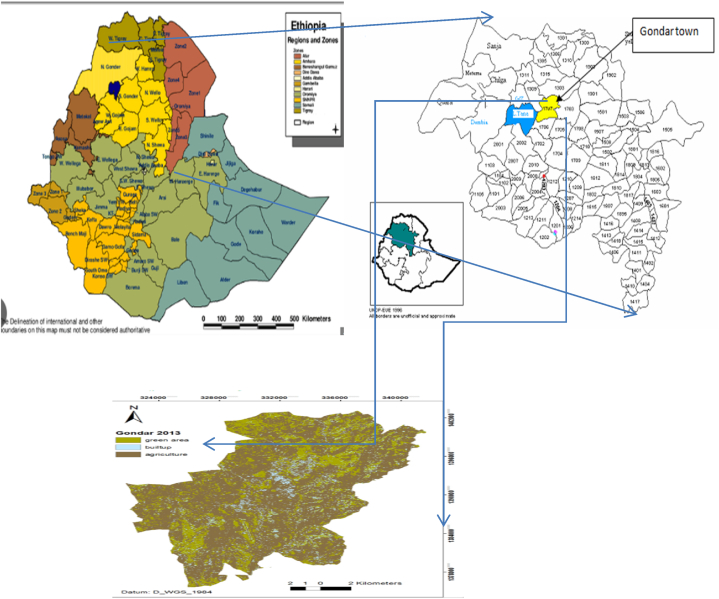
Map of the area.

The study was carried out in Gondar town, situated in the central Gondar zone within the Amhara Regional State in Northwest Ethiopia. Gondar town's geographical coordinates are 12°36′N 37°28′E, with an altitude of 2133 m above sea level [28] as shown in Fig. 1. It is positioned 739 km away from Addis Ababa, the capital city of Ethiopia [29]. The town experiences an average annual temperature of 20 °C, with rainfall ranging from 880 to 1172 mm. The area is characterized by two distinct seasons: the dry season, spanning from October to May, and the wet season, lasting from June to September. The town's climate falls under the classification of "Weynadega" [30]. Furthermore, it has a mixed livestock rearing system, 413000 individuals, and an estimated 5000–10,000 dogs [7].

### 2.2 Study populations

This study involved domestic dogs located in Gondar town as study animals. The main focus was on determining the presence or absence of gastrointestinal nematode parasites in the dogs. Additionally, the predictable factors influencing infections by gastrointestinal nematode worms were species, sex, age, breed, housing conditions, and body condition scores [3,26,31,32]. The different varieties of dogs within the breed were categorized based on their historical background and physical characteristics, such as coat type, size, and overall build. The age of the dogs was classified as young (up to one year) and adult (above one year) following Tizard's guidelines [33]. Furthermore, their body condition scores were recorded as poor, medium, and good according to the Baldwin guidelines [34]. The housing system also distinguished between stray dogs, which were allowed to roam during certain times of the day, and confined dogs that were not permitted to leave their owner's premises.

### 2.3 Study designs

A cross-sectional study design was conducted from December 2022 to April 2023 to determine the prevalence of GIT nematode parasites in Dogs and associated risk factors in the study area.

### 2.4 Sample size determination and sampling methods

The Thrusfield formula [35] was used to calculate the sample size. It was determined based on 14.7 % of previous prevalence studies in the research area [7], with a 5 % absolute accuracy at a 95 % confidence interval (CI). The formula used was: N = [1.96^2^Pexp (1-Pexp)]/d^2^.Where, N = the number of required samples.

Pexp = anticipated prevalence (>50 %)

d = desired absolute precision level at 95 % confidence interval (=0.05)

As a result, the study had a calculated sample size of 193. To increase the precision, 204 dog stool specimens were collected along with a 5 % non-response rate. The limited collection of samples was due to constraints in time, space, and access to dog cases during the study period. Furthermore, some participants were unwilling to collect dog stool samples in their homes. Some alert dogs were also uncomfortable taking feces samples. A purposive sampling strategy was utilized to choose 204 dogs located in the study area to determine the GIT nematode worms’ presence and their associated risk factors.

### 2.5 Sample collection methods

Fresh fecal samples were obtained directly from the rectum of each dog involved in the study. The samples were subsequently placed in sterilized collection bottles. Each bottle was labelled with essential information, including the dog's age, sex, breed, housing conditions, body condition, and the date of collection. Subsequently, the samples were stored in an ice box for transportation to the Veterinary Parasitology Laboratory at the University of Gondar. They were stored at a temperature of 4 °C in a refrigerator until analysis. Direct smear and flotation techniques were also used to identify parasite eggs in dogs.

#### 2.5.1 Direct smear

A minute quantity of feces was placed on a slide and mixed with one droplet of distilled water and then covered with a coverslip for a direct fecal smear. The slide was thoroughly inspected to identify any nematode parasite eggs. Subsequently, the eggs were identified by their morphological characteristics [36,37].

#### 2.5.2 Flotation techniques

The flotation solution was prepared by blending 400 g of sodium chloride (NaCl) into 1000 mL of distilled water and agitating until the salt crystals dissolved [38,39]. The process began by transferring about 3 g of feces and flotation solution into a paper cup. Next, the solution was thoroughly mixed using a tongue depressor and filtered through a metal tea strainer into a second paper cup. A glass coverslip was placed over the meniscus, and after 10–20 min, it was removed to allow the nematode egg concentration to stabilize. Following this, the sample was placed onto a glass microscope slide for microscopic analysis. To avoid bubble formation, the samples were swiftly placed on the microscope slides and examined under the microscope [[36], [37], [38], [39]]. The principle allowed for the eggs to float to the surface of the solution of sodium chloride with standard specific gravity (1.20), which concentrates at the top and leaves debris lower down [40,41]. However, the higher the specific gravity of the solution, the more eggs of various types will float [42].

### 2.6 Data analysis methods

Data was entered and analyzed using SPSS version 25. Descriptive statistics (frequencies and percentages) were computed. Chi-square (X^2^) was used to assess the difference in positive frequency of the GIT nematode occurrence between the associated risk factors. Moreover, univariate logistic regression analysis was used to identify some of the potential risk factors associated with the GIT nematode occurrence. The strength of associations was also calculated using an odds ratio (OR) at a 95 % confidence interval (CI) and a *P*-value ≤0.05 was seen as statistically significant. Multivariate logistic regression also checked the confounding of the significant risk factors in univariate logistic regressions (*P-value* ≤ 0.05).

## 3 Findings

### 3.1 Overall prevalence of GIT nematode parasites in dogs

The overall prevalence of GIT nematode parasites in dogs was 47 (23.03 %) from the total 204 Dog fecal samples. The most frequently found GIT nematode parasites were *Ancylostoma* (9.31 %) followed by *Toxocara* (5.39 %), *Trichuris vulpis* (4.41 %) and *Strongyloides* (3.92 %) as shown in Table-1.

**Table 1:** The overall occurrence of gastrointestinal nematode parasites in Dogs (N = 204).

Species	Frequency	Percentage (%)	(95 % CI)
*Anchylostoma*	19	9.31 %	(5.4–13.7)
*Toxocara*	11	5.39 %	(2.5–8.8)
*Trichuris*	9	4.4 %	(2–7.4)
*Strongyloides*	8	3.92 %	(1.5–6.9)
**Total**	**47**	**23.03 %**	**(17.6**–**29.4)**

### 3.2 Distribution of dogs associated potential risk factors

Male dogs had a higher exposure rate (84.3 %) compared to female dogs (15.7 %). Adult dogs (63.7 %) were also more exposed to these parasites compared to young pups (36.3 %). Furthermore, constrained housing (84.8 %) and local breed (94.1 %) of dogs showed higher records of these parasites. The most common body condition scores (BCS) for dogs were good (86.8 %), followed by medium (10.8 %) and poor (2.4 %) (Table-2).

**Table 2:** Distribution of indicator variables for GIT nematodes of dogs (N = 204).

Variable	Category	Total examined	Percentages
Sex	Female	32	15.7 %
	Male	172	84.3 %
Age	Adult	130	63.7 %
	Young	74	36.3 %
Housing system	Confined	173	84.8 %
	Stray	31	15.2 %
Breed	Local	192	94.1 %
	Cross	12	5.9 %
BCS	Good	177	86.8 %
	Medium	22	10.8 %
	Poor	5	2.4 %
Total		204	100 %

### 3.3 The occurrence of GIT nematode parasites of dogs within risk factors

The study examined the prevalence of gastrointestinal nematodes in dogs, focusing on various risk factors including sex, age, body condition scores, housing conditions, and breed type. Statistically significant differences were observed between the presence of these parasites and the assessed variables, except breed groups (*P-value* > 0.05). The categories with the highest percentages of infested dogs included females (50 %), younger dogs (52.7 %), those managed in stray housing conditions (90.32 %), crossbreeds (41.6 %), and dogs in poor body condition (60 %) as shown in Table 3.

**Table 3:** The prevalence of GIT nematodes of dogs with associated risk factor.

Variable	Category	Total examined	No positive (%)	*X*^*2*^	*P-value*
Sex	Female	32	16 (50 %)	14.56	0.000
	Male	172	31 (18.02 %)		
Age	Adult	130	8 (6.15)	9.79	0.002
	Young	74	39 (52.7 %)		
Housing system	Confined	173	19 (10.98 %)	93.33	0.000
	Stray	31	28 (90.32 %)		
Breed	Local	192	42 (21.87 %)	2.49	0.114
	Cross	12	5 (41.66 %)		
BCS	Good	177	33 (18.64 %)	14.79	0.001
	Medium	22	11 (50 %)		
	Poor	5	3 (60 %)		
Total		204	47 (23.03 %)		

The presence of GIT nematode species such as *Aychylostoma*, *Toxocara*, *Tricuris*, and *Strongyles* was found to have significant statistical associations with risk factors such as sex, age, housing system, and body condition. Female and young dogs were more likely to be infected with these GIT nematode species. Furthermore, stray dogs were more prone to *Toxocara* and *Strongyle* infections, while confined dogs were more affected by *Ancylostoma* and *Tricuris*. Additionally, *Ancylostoma* and *Strongyle* infections were more prevalent in poor-condition dogs, while *Tricuris* infections were more common in medium-condition dogs, and *Toxocara* infections were more frequent in good-condition dogs, as shown in Table 4.

**Table 4:** The occurrence of GIT nematode species of dogs with their significant risk factors.

GIT nematode	RF	Categories	N (%)	X^2^ (*P*-value)
Anchylostoma	Sex	Female	5 (15.6)	0.001
		Male	14 (8.1)	
	Age	Young	15 (11.5)	0.020
		Adult	4 (5.4)	
	Housing	Confined	18 (58.1)	0.001
		Stray	1 (0.6)	
	BCS	Poor	3 (13.6)	0.001
		Medium	0 (0)	
		Good	16 (9.0)	
Toxocara	Sex	Female	2 (6.3)	0.001 0.020 0.001
		Male	9 (5.2)	
	Age	Young	9 (6.9)	
		Adult	2 (2.7)	
	Housing	Confined	0 (0)	
		Stray	11 (6.4)	
	BCS	Poor	1 (4.5)	0.001
		Medium	0 (0)	
		Good	10 (5.6)	
Tricuris	Sex	Female	7 (21.9)	0.001
		Male	2 (1.2)	
	Age	Young	9 (6.9)	0.020
		Adult	0 (0)	
	Housing	Confined	9 (29.0)	0.001
		Stray	0 (0)	
	BCS	Poor	6 (27.3)	0.001
		Medium	3 (60.0)	
		Good	0 (0)	
Strongyloides	Sex	Female	2 (6.3)	0.001 0.020 0.001
		Male	6 (3.5)	
	Age	Young	6 (4.6)	
		Adult	2 (2.7)	
	Housing	Confined	1 (3.2)	
		Stray	7 (4.0)	
	BCS	Poor	1 (4.5)	0.001
		Medium	0 (0)	
		Good	7 (4.0)	
Total			47 (23.03)	

The univariable logistic regression analysis indicated a significant association between sex, age, body condition, and housing systems and the overall prevalence of parasites (*P-value* ≤ 0.05). However, these risk factors did not exhibit confounding effects or multicollinearity in the multivariate logistic regression analysis (*P-value* > 0.05). Female dogs had a 4.5 times higher likelihood (COR: 4.55; 95 % CI: 2.05–10.07) and young dogs had a 3.5 times higher likelihood (COR: 3.5; 95 % CI: 1.55–8.06) of being exposed to these parasites compared to male and adult dogs, respectively. In terms of body condition scores, poor-condition dogs were 6.5 times more likely (COR: 6.55; 95 % CI: 0.06–0.56) and medium-condition dogs were 0.5 times more likely (COR: 1.5; 95 % CI: 0.26–0.99) to be exposed compared to dogs with good body conditions as shown in Table 5.

**Table 5:** Univariable and multivariable logistic regression analysis of risk factors.

Variable	Category	COR(95%CI)	*P-value*	AOR(95 % CI)	*P-value*
Sex	Female	1		1	0.182
	Male	4.55(2.05–10.07)	0.000	2.21(0.69–7.08)	
Age	Adult	1		1	0.086
	Young	3.54(1.55–8.06)	0.003	2.68(0.87–8.28)	
Housing system	Confined	1		1	0.000
	Stray	75.65(20.98–27.2)	0.000	59.12(15.43–226.39)	
Body condition	Good	1		1	0.674
	Medium	1.5(0.26–0.9 9)	0.050	0.48(0.01–14.35)	
	Poor	6.55(0.06–0.56)	0.003	1.02(0.04–23.92)	0.989

NB: COR: Crude odds ratio; AOR: Adjusted odds ratio; CI: Confidence interval

## 4 Discussions

The overall prevalence of GIT nematode parasites of dogs in this study was found that 23.03 % (95 % CI = 17.6–29.4). This finding has coincided with studies conducted in different parts of the world [16,[43], [44], [45], [46]]. The use of the same methodology and parasitological techniques could account for these findings. However, it was lower than the previous studies from Ethiopia [4,5,23,24] with the overall prevalence of 94.6 %, 50.9, 59 % and 56 %, respectively, and elsewhere in the globe with 56 % in Nigeria [47] and 33.3 % in Rwanda [48]; 31.75 % in Brazil [49]; 33.6 % in Virginia of the USA [50]; 34.68 % in Van province [51]; 35.9 % in Egypt [52]; 58.3 % in Serbia [53] and 57.2 % in Portugal [54]. On the other hand, this finding was also higher than the findings of 14.7 % in Gondar town [7]; 4 % in Spain [55] and 11.5 % in Poland [56]. The variations in these findings may be due to differences in the research methods, the procedures for parasitological techniques, the environmental conditions for the parasite's survival, the availability of veterinary services and the level of awareness about dog care [3,21,57]. Moreover, the nematode parasite occurrence differs significantly across geographic regions due to the specific GIT nematode, animal species, and local environmental factors like humidity, temperature, rainfall, vegetation, and management practices [22]. The variation in the presence of parasites could be also adversely affected by acidic and alkaline soil conditions [32]. Inadequate hygiene practices and substandard housing conditions, such as lack of confinement indicated the variation in the occurrence of these parasites among dogs [58]. The variation is also associated with environmental contamination caused by dog feces, which serves as a considerable source of infection for both livestock and humans [15,56].

The most gastrointestinal parasites identified in this study were *Ancylostoma* (9.31 %) followed by *Toxocara* (5.39 %), *Strongyloides* (4.41 %) and *Tricuris* (3.92 %). These highest findings of *Ancylostoma* were agreed from various studies in Ethiopia [5,24,59]; Nigeria [47]; Rwanda [48]; Morocco [60]; Brazil [49]; USA [50]; Poland [17] and Central Oklahoma [44]. This observed frequency of *Ancylostoma* (9.31 %; 95 % CI = 5.4–13.7) has also coincided with 5.6 % in Van province [61]; 8 % in Poland [17] and 9.8 % in Northern Greece [45]. However, it was lower than the previous reports in Ethiopia [4,5,24]; Morocco [60]; Rwanda [48]; Brazil [49]; Virginia of the USA [50] and Portugal [54]. On the other hand, this finding was higher than the previous reports of 4 % in Spain [55]; and 4.6 % in Ethiopia [7]. The implication is that dogs commonly carry most zoonotic nematode worms, such as *Anchylosoma* and *Toxocara*, which can be transmitted through the ingestion of infective eggs and the ingestion or skin penetration of infective larvae [62].

In the case of *Toxocara* occurrence (5.39 %); 95 % CI: 2.5–8.8) also coincided with Spain (6.3 %) [55] and 3.2 % in Rwanda [48] and It was also lower than from Ethiopia [4,14,18,59,63] and elsewhere the world [43,45,49,50,52,60]. The *Strongyloides* and *Tricuris* occurrence were also in line with 3.92 % in Ethiopia [25]. These findings were also higher than the previous reports of 0.97 % [24]; 1.31 % [16] and 1.66 % [54]. These above-mentioned finding variations could be attributed to the study methodology differences, management practices, health care systems, levels of environmental contamination by infectious stages, and exposure to natural infections more frequently than owning dogs. *Ancylostoma* caninum is the most widespread of all hookworm species and it parasitizes dogs throughout the tropics and subtropics [64]. The survival and development parasites of ova are affected by both temperature and the availability of moisture [65]. *Toxocara* is more prevalent in hot, humid regions where eggs are kept viable in the soil [66].

The occurrence of the GIT nematode of infested dogs has a statically significant association between all observed risk factors such as sex, age, body condition scores and housing systems (*P-value* < 0.05) except dog breeds (*P-value* >0.05). These findings were in line with the previous reports from Ethiopia [4,14] and in the World including in Brazil [49]. Nevertheless, these findings disagreed with the earlier reports of Gugsa [63], Tamerat [18], Savilla [50], Al-Sabi [43], and Karakus and Denizhan [51] who stated that the occurrence of intestinal nematodes in dogs was not statically significant with sex, age and breeds of the dogs.

In the case of sex, female dogs were 4.5 times more likely exposed to these parasites than male dogs (COR (95%CI): 4.55 (2.05–10.07)). More females (50 %) were observed than males (18.02 %). This finding was supported by the previous reports [5,25]. On the contrary, this finding is contradicted by the various findings in Ethiopia [4,23,24]; Rwanda [48]; Nigeria [47] and Pakistan [16]. These sex-wise variations might be attributed to stress, such as pregnancy and lactation in female dogs more vulnerable to various nematode infections. Pregnant bitches serve as the somatic larvae cross the placental barrier to infect the neonates [67]. Conversely, male dogs are typically favoured by many for their assertive nature, making them suitable as protectors. Female dogs are often avoided because of the inconvenience of a female in heat-attracting packs of unfixed males [68].

The age wise GIT nematode occurrences showed that young dogs (52.7 %) were 3.5 times more likely exposed to these parasites than adult dogs (6.15 %) (COR (95 % CI): 3.54 (1.55–8.06)). This finding was supported from the various previous studies [46,47,54,59]. However, In Ethiopia [5,23,24] and Pakistan [16] who were contradicted the significant association of the prevalence of GIT nematodes between the age groups. These variations might be attributed to study methodology differences and agroeclogical and climatic conditions. These young dogs have not developed an immune response, thus it is a major source of soil contamination and transmission of infection to humans [69]. Poor was 6.5 times and medium body conditions were also 0.5 times more exposed than the dogs had good body condition scores (COR: 6.55; 95 % CI: 0.06–0.56); (COR: 1.5; 95 % CI: 0.26–0.9 9), respectively. GIT nematode was highly infested in poor and medium body condition scores than in dogs that had good body conditions. This finding was agreed with the reports of Merga and Sibhat [14]. On the other hand, the finding contradicted the reports of Dubie et al. [23]. These variations could be attributed to the poor and medium body conditions animals had reduced immunity predisposed to various parasitic diseases than the dogs had good body conditions [70].

Regard to the management system indicated that stray dogs (90.32 %) were 3.5 times more likely exposed to these parasites than confined dogs (10.98 %) (COR: 75.65; 95 % CI: 20.98:27.2). This finding was agreed with various earlier studies in Ethiopia [14] and Portugal [54]. Nevertheless, these findings were contradicted by the finding of Asmara and Mekuria [71] who stated closing housing (64.9 %) dogs were more observed than free-controlled dogs (41.96 %). Besides, various reports stated that housing management of dogs and their infestation had not statically significant [24,48]. These variations might be attributed to freely moving and scavenging dogs around high contact with contaminated environments to expose these parasites [72].

## 5 Conclusion and recommendations

The study illustrated that the existence of these nematode worms threatens the health of affected dogs and has public health risks as they coexist in shared environments. *Ancylostoma* had the highest prevalence in this study, and it is of zoonotic significance. The presence of these parasites had a significant impact on female and younger stray dogs that exhibited poor body condition scores. Therefore, it is important to implement appropriate preventive measures to halt the transmission of these nematode worms. Dog owners should be informed about the potential for dogs to transmit zoonotic parasites to humans. Further research on the detailed epidemiology of helminth infections in dogs should be conducted.

## CRediT authorship contribution statement

**Tsedalu Yirsa:** Conceptualization, Investigation, Writing – original draft. **Amare Bihone:** Supervision, Software, Methodology, Project administration. **Getenew Teshager:** Investigation, Data curation, Methodology. **Yimer Muktar:** Software, Formal analysis, Writing – review & editing. **Abebe Berihun:** Visualization, Methodology, Validation.

## Ethics approval statements

All procedures and animal care adhered to the guidelines established by the Federation of Animal Science Societies (FASS) [73] guidelines. The study was conducted in Gondar town and received ethical clearance from the Institutional Review Board (IRB) of Woldia University, Ethiopia (WDU/IRB019/01/2022). Additionally, oral informed consent was obtained from each dog owner prior to participation. The objectives and significance of the research were clearly explained to the participants by the investigator. No known risks or discomforts were associated with the collection of stool specimens from the dogs.

## Consent for publication

Not applicable.

## Data availability statement

The data can be obtained from the corresponding author upon request of this email (tsedyirsa@gmail.com), as it is subject to ethical restrictions.

## Funding

We acknowledge the University of Gondar for providing laboratory reagents and equipment support for this study. We also acknowledged the owners of dogs who lived in Gondar town to restrain and allow feces sample collection.

## Declaration of competing interest

The authors declare the following financial interests/personal relationships which may be considered as potential competing interests:Tsedalu Yirsa reports equipment, drugs, or supplies was provided by University of Gondar College of Medicine and Health Sciences. Tsedalu Yirsa reports a relationship with Woldia University that includes: employment. Tsedalu Yirsa has patent no issued to no. No more conflict of interest exists. If there are other authors, they declare that they have no known competing financial interests or personal relationships that could have appeared to influence the work reported in this paper.

## List of abbreviations

A.canium: Anchylostoma canium
CI: Confidence interval
EPG: Egg per gram
GIT: Gastrointestinal
HSG: High specific gravity
Pexp: Expected prevalence
SPP: Species
T.cani: Toxocara cani
